# Bayesian variable selection in joint modeling of longitudinal data and interval-censored failure time data

**DOI:** 10.21203/rs.3.rs-4254893/v1

**Published:** 2024-04-18

**Authors:** Yuchen Mao, Lianming Wang, Xiaoyan Lin, Xuemei Sui

**Affiliations:** 1 Department of Statistics, University of South Carolina, Columbia, SC, USA.; 2 Department of Exercise Science, Arnold School of Public Health, University of South Carolina, Columbia, SC, USA.

**Keywords:** Bayesian Lasso, interval-censored data, joint modeling, longitudinal data, variable selection

## Abstract

Joint modeling of longitudinal data and survival data has gained great attention in the last two decades. However, most of the existing studies have focused on right-censored survival data. In this article, we study joint analysis of longitudinal data and interval-censored survival data and conduct Bayesian variable selection in this framework. A new joint model is proposed with a shared frailty to characterize the dependence between the two types of responses, where the longitudinal response is modeled with a semiparametric linear mixed-effects submodel and the survival time is modeled by a semiparametric normal fraility probit sub-model. Several Bayesian variable selection approaches are developed by adopting Bayesian Lasso, adaptive Lasso, and spike-and-slab priors in order to simultaneously select significant covariates and estimate their effects on the two types of responses. Efficient Gibbs samplers are proposed with all unknown parameters and latent variables being sampled directly from well recognized full conditional distributions. Our simulation study shows that these methods perform well in both variable selection and parameter estimation. A real-life data application to joint analysis of blood cholesterol level and hypertension is provided as an illustration.

## 1 Introduction

Longitudinal data commonly arise in epidemiological, social-economic, and medical studies where participants are measured multiple times at discrete time occasions during the study, and the research interests are to estimate the mean trajectory of the response over time and to assess the effects of potential covariates. Interval-censored data also arise in such studies, but the response variable of interest is the failure time of a certain event such as a disease, or death. Since the event status is only evaluated at the examination times, the exact time of the failure event is never observed but is only known to fall within some interval formed by two adjacent examination times with failure status being changed. In this article, we study joint modeling of longitudinal data and interval-censored data by borrowing information between correlated longitudinal and survival responses particularly in the case that there are many potential covariates available for both types of responses.

Joint modeling of longitudinal and survival responses has been popular in the statistical literature in the last two decades because it provides more efficient parameter estimation than separate univariate analyses (Elashoff et al., 2008; Diggle et al., 2008; Asar et al., 2015; Chen et al., 2018). Most of the existing works on joint modeling have focused on the joint analysis of longitudinal data and right-censored survival data, where the failure time is either exactly observed or right-censored for all subjects in the study. The research on joint analysis of longitudinal data and interval-censored data is relatively sparse due to the more complex data structure but has drawn more attention in recent years. Available research works on this topic include Lee et al. (2011), Gueorguieva et al. (2012), Ye et al. (2015), Rouanet et al. (2016), Chen et al. (2018), Grand et al. (2015), Yi et al. (2020), Weng et al. (2022), and Yi et al. (2022). It is worth noting that all of these approaches were developed from frequentist’s perspectives, and the development of Bayesian research on this topic is clearly needed.

In real life longitudinal studies, there are usually many demographic variables and health-related characteristics available for participants when examined multiple times. Identifying significant risk factors for the responses of interest is naturally an important and challenging research topic. Variable selection methods have been developed to tackle this problem, and existing techniques include forward selection, backward elimination, stepwise selection, and model comparisons using Bayes factor or some other information criteria (Akaike, 1974; Hocking, 1976; Lee and Tang, 2006). These traditional methods are usually computationally expensive and not well suitable to deal with models with a large number of covariates. In response to that, some regularization approaches, such as the least absolute shrinkage and selection operator (Lasso), the adaptive Lasso, and the smoothly clipped absolute deviation (SCAD), have been developed for variable selection (Tibshirani, 1996; Fan and Li, 2001; Zou, 2006). In particular, Tibshirani (1996) showed that Lasso does parameter estimation and variable selection simultaneously based on the shrinkage property of the $L_{1}$ penalty and suggested from a Bayesian perspective that Lasso estimates can be interpreted as the posterior mode when the regression parameters are assigned with independent and identical Laplace (i.e., double-exponential) priors. Park and Casella (2008) developed an efficient Gibbs sampler for the Bayesian Lasso by exploiting a hierarchical representation of the full model. Leng et al. (2014) further proposed Bayesian adaptive Lasso that allowed different penalties for different regression parameters. The spike-and-slab method is also widely adopted in variable selection (Mitchell and Beauchamp, 1988; George and McCulloch, 1997; Ishwaran and Rao, 2005). O’Hara and Sillanpää (2009) also provided a comprehensive review and comparison of the above-mentioned Bayesian variable selection methods in the conventional linear regression settings.

There are only a few works in the literature on variable selection in the context of joint modeling of longitudinal data and survival data due to the great complexity in such data. For joint analysis of longitudinal and right-censored survival data, Tang et al. (2017) proposed a longitudinal sub-model with a normal mixture for the error distribution and developed a Bayesian Lasso combined with the use of penalized splines, and Xie et al. (2020) developed an adaptive group Lasso to select both time-varying and time-invariant effects in both longitudinal and survival submodels. Yi et al. (2022) is the only available work to date on variable selection in joint modeling of longitudinal and interval-censored data, and they developed Lasso and adaptive Lasso based on a Monte Carlo expectation-maximization (MCEM) algorithm in their estimation approach. All these studies adopted the frailty proportional hazards (PH) model for the survival time in their modeling.

The objective of this article is to develop efficient Bayesian variable selection approaches to simultaneously select covariates and estimate their effects in joint modeling of longitudinal and interval-censored survival data. The proposed model contains a semiparametric mixed-effects submodel for the longitudinal response, a frailty semiparametric probit submodel for the survival time (Lin and Wang, 2010; Wu and Wang, 2019), and a shared random effect to characterize the association between the longitudinal and survival responses. We explore Bayesian Lasso, adaptive Lasso, and spike-and-slab methods in this joint modeling framework in a unified manner with different prior specifications for the regression coefficients. Our proposed Gibbs samplers are computationally efficient because the full conditional distributions of all unknown parameters and latent variables take standard forms such as normal, gamma, and inverse-Gaussian distributions.

The remainder of the article is organized as follows. In Section 2, we present our proposed joint model for longitudinal and interval-censored survival responses as well as the observed likelihood. In section 3, a unified Bayesian estimation approach is developed for the joint analysis with the incorporation of variable selection techniques, resulting in four computationally efficient Gibbs samplers. Section 4 evaluates and compares the proposed methods through a simulation study, and Section 5 presents a real-life application as an illustration of the proposed methods. Some concluding remarks are given in Section 6.

## 2 Joint model of longitudinal and survival responses

### 2.1 The proposed joint model

For a typical longitudinal study, let $t_{i1}<t_{i2}<\cdots <t_{im_{i}}$ denote the sequence of pre-specified examination times for the ith individual. Let $T_{i}$ denote the failure time of interest for subject $i,X_{i}$ a vector of covariates for subject i, and $y_{ij}=y\left(t_{ij}\right)$ the observed value of the longitudinal response at $t_{ij}$ for $j=1,\ldots ,m_{i}$ and i=1,…,n. Under the longitudinal study design, the statuses of failure are only examined at the observation times $t_{ij}$’s, so $T_{i}$ is not directly observed but is known to fall within some interval $\left(L_{i},R_{i}\right)$ that is formed by two closest examination times (including 0 and ∞) with changed statuses. We say that $T_{i}$ is left-censored if $0=L_{i}<R_{i}<\infty$, right-censored if $0<L_{i}<R_{i}=\infty$, and strictly interval-censored otherwise.

We consider the following semiparametric mixed effects model for the longitudinal response,

(1) $$y_{ij}=\mu \left(t_{ij}\right)+X_{i}^{\prime }\theta +\xi_{i}+u_{i}+\epsilon_{ij},$$

where μ(⋅) is the unspecified baseline mean function over time, θ is the vector of fixed covariate effects on the response, $\xi_{i}$ is a frailty to introduce within-subject association among the longitudinal measurements for subject $i,u_{i}$ is a frailty shared by both the longitudinal and survival responses for subject i, and $\epsilon_{ij}$’s are independently and identically distributed random errors. It is assumed that $u_{i}\sim N\left(0,\sigma_{u}^{2}\right),\xi_{i}\sim N\left(0,\sigma_{\xi }^{2}\right)$ in our model, and $\epsilon_{ij}\sim N\left(0,\sigma_{\epsilon }^{2}\right)$, and all these frailties and random errors are mutually independent.

The following semiparametric normal frailty probit model is assumed for the event time $T_{i}$ conditional on the frailty $u_{i}$,

(2) $$F\left(t|X_{i},u_{i}\right)=\Phi \left\{\alpha (t)+X_{i}^{\prime }\beta +u_{i}\right\},$$

where $F\left(\cdot |X_{i},u_{i}\right)$ is the conditional cumulative distribution function (CDF) of $T_{i}$ given covariates $X_{i}$ and frailty $u_{i},\Phi (\cdot )$ is the CDF of a standard normal distribution, β is the vector of covariate effects on the event time, and α(⋅) is an unspecified increasing function with ${\text{lim}}_{t\to 0}\alpha (t)=-\infty$ and ${\text{lim}}_{t\to +\infty }\alpha (t)=+\infty$.

We assume that the longitudinal and survival responses are conditionally independent given the shared random effect. The shared random effect induces statistical dependence between the longitudinal response and the survival response. The statistical association between $y_{ij}$ and $T_{i}$ under the proposed joint model can be summarized in terms of Kendall’s τ in the following simple explicit form,

(3) $$\tau =\frac{2}{\pi }{\text{sin}}^{-1}\left(\rho \right),$$

where ρ is the Pearson’s correlation coefficient between longitudinal response $y_{ij}$ and the transformed failure time $\alpha \left(T_{i}\right)$ taking the following form

$$\rho =\frac{-\sigma_{u}^{2}}{\sqrt{\left(1+\sigma_{u}^{2}\right)\left(\sigma_{\epsilon }^{2}+\sigma_{\xi }^{2}+\sigma_{u}^{2}\right)}}.$$

The expression of Kentall’s τ in (3) is obtained based on the relationship between Pearson’s correlation coefficient and Kentall’s τ under bivariate normal distribution (Kruskal, 1958; Hougaard, 2000). This statistical association in terms of Kendall’s τ is easy to calculate with a simple explicit form. The expression in (3) also suggests that the statistical association does not depend on any covariates but rather depends on the variances of the frailties and random errors involved in the joint model. Kendall’s τ in equation (3) is always negative under the proposed joint model, and this means that larger values of the longitudinal response correspond to smaller values of the survival time, i.e., a higher risk of survival event. If the statistical association is in an opposite direction in a real life study, one can modify model (2) by simply replacing $u_{i}$ with $-u_{i}$ for the application.

Under the proposed joint model and the aforementioned assumptions, the observed likelihood takes the following form

(4) $$L_{obs}=\prod_{i=1}^{n}\iint \;\left\{\prod_{j=1}^{m_{i}}g\left(y_{ij}|X_{i},u_{i},\xi_{i}\right)\right\}\left\{F\left(R_{i}|X_{i},u_{i}\right)-F\left(L_{i}|X_{i},u_{i}\right)\right\}\;\sigma_{u}^{-1}\phi \left(\sigma_{u}{\;}^{-1}u_{i}\right)\sigma_{\xi }^{-1}\phi \left(\sigma_{\xi }^{-1}\xi_{i}\right)du_{i}d\xi_{i},$$

where $g\left(y_{ij}|x_{i},u_{i},\xi_{i}\right)$ is the normal density function of $y_{ij}$ given covariates $X_{i}$ and frailties $u_{i}$ and $\xi_{i}$ based on equation (1), and ϕ(⋅) is the probability density function of the standard normal distribution. The unknown parameters involved in this likelihood include two baseline functions μ(⋅) and α(⋅), two sets of regression parameters θ and β, and three variance parameters for the frailties and random errors. Since the integrals in this observed likelihood do not have a closed form, estimation based on this likelihood directly is challenging from both frequentist’s and Bayesian perspectives.

### 2.2 Modeling unspecified functions with splines

Another complication in the estimation is that the two unknown baseline functions μ(⋅) and α(⋅) are both infinitely dimensional. To tackle this problem, spline methods are adopted so that one only needs to estimate a finite number of parameters for these two functions. Specifically, we use a linear combination of M-splines to approximate μ(⋅) as follows,

$$\mu \left(t\right)=\sum_{h=1}^{\tilde{K}}\eta_{h}{\tilde{M}}_{h}\left(t\right),$$

where ${\tilde{M}}_{h}$’s are M-spline basis functions (Ramsay, 1988) and $\eta_{h}$’s are unconstrained spline coefficients. The values of the M-spline basis functions ${\tilde{M}}_{h}$’s depend on the chosen knots and degree $\tilde{d}$. The knots are typically an increasing sequence within the data range. The degree $\tilde{d}$ can be 1, 2, or 3, which corresponds to linear, quadratic, or cubic M-spline basis functions. Let $\tilde{J}$ denote the number interior knots, and the number of M-spline basis functions is determined as $\tilde{K}=\tilde{J}+\tilde{d}+1$.

We approximate α(⋅) in a similar manner. Since α is non-decreasing, following Lin and Wang (2010) we adopt the following monotone spline expression for modeling α(⋅),

$$\alpha (t)=r_{0}+\sum_{k=1}^{K^{*}}r_{k}b_{k}(t)$$

where $b_{k}$’s are I-spline basis functions (Ramsay, 1988), which are integrated functions of corresponding M-splines, $r_{k}$’s are the spline coefficients that are constrained to be nonnegative, and $r_{0}$ is an unconstrained intercept. These I-spline basis functions are nondecreasing piecewise polynomials, taking 0 in the first stage, increasing up to 1 in the second stage, and staying in plateau of 1 in the third stage. The number of I-spline basis functions $K^{*}$ is determined by the number of interior knots $J^{*}$ and the degree $d^{*}$ for the associated M-spline functions, i.e., $K^{*}=J^{*}+d^{*}+1$. The construction and evaluation of M-spline and I-spline functions can be performed directly using R package splines2 (Wang and Yan, 2021).

To ensure enough flexibility, one can use many knots in specifying the splines for modeling an unknown function. A potential problem of using a large number of knots is overfitting in addition to the computation burden. To tackle these problems, shrinkage priors can be used for the spline coefficients to penalize large values and shrink the coefficients of the unnecessary basis functions towards zero (Lin and Wang, 2010; Wang and Dunson, 2011).

## 3 Data augmentation and prior specification

### 3.1 Data augmentation

The complicated form of the observed likelihood make it a great challenge for posterior computation no matter what priors are adopted for the parameters. To develop an efficient Bayesian estimation approach, we first propose a two-stage data augmentation that leads to a complete data likelihood with a simple and nice form for posterior computation.

In the first stage, we consider the following conditional likelihood given $u_{i}$’s and $\xi_{i}^{\prime }$’s,

(5) $${ℒ}_{1}=\prod_{i=1}^{n}\left[\left\{\prod_{j=1}^{m_{i}}g\left(y_{ij}|X_{i},u_{i},\xi_{i}\right)\right\}\left\{F\left(R_{i}|X_{i},u_{i}\right)-F\left(L_{i}|X_{i},u_{i}\right)\right\}\frac{1}{\sigma_{u}}\phi \left(\frac{u_{i}}{\sigma_{u}}\right)\frac{1}{\sigma_{\xi }}\phi \left(\frac{\xi_{i}}{\sigma_{\xi }}\right)\right].$$

The second-stage of our data augmentation is motivated by Lin and Wang (2010) to simplify the likelihood contribution from the interval-censored survival data in the observed likelihood (4). For convenience, let $\delta_{i1},\delta_{i2}$, and $\delta_{i3}$ be the censoring indicators of $T_{i}$ being left-, interval-, and right-censored, respectively. Since only one type of censoring occurs for each subject, there is a constraint $\delta_{i1}+\delta_{i2}+\delta_{i3}=1$ for each i. With these notations, we introduce a normal latent variable

$$z_{i}\sim N\left(\alpha \left(t_{i}\right)+X_{i}^{\prime }\beta +u_{i},1\right)$$

subject to a constraint $z_{i}\in C_{i}$ for subject i, where $t_{i}=R_{i}1_{\left(\delta_{i1}=1\right)}+L_{i}1_{\left(\delta_{i1}=0\right)}$, and the constrained space $C_{i}$ takes the form of (0,∞) if $\delta_{i1}=1,\left(\alpha \left(L_{i}\right)-\alpha \left(R_{i}\right),0\right)$ if $\delta_{i2}=1$, and (-∞,0) if $\delta_{i3}=1$ for each i.

Based on this data augmentation, the complete data likelihood given all $z_{i}$’s, $u_{i}$’s and $\xi_{i}$’s takes the following form

(6) $${ℒ}_{C}=\prod_{i=1}^{n}\left[\left\{\prod_{j=1}^{m_{i}}g\left(y_{ij}|X_{i},u_{i},\xi_{i}\right)\right\}\phi \left(z_{i}-\alpha \left(t_{i}\right)-X_{i}^{\prime }\beta -u_{i}\right)1_{C_{i}}\left(z_{i}\right)\frac{1}{\sigma_{u}}\phi \left(\frac{u_{i}}{\sigma_{u}}\right)\frac{1}{\sigma_{\xi }}\phi \left(\frac{\xi_{i}}{\sigma_{\xi }}\right)\right].$$

It is straightforward to show that integrating out all the $z_{i}$’s in the complete likelihood (6) leads to the conditional likelihood (5). This complete data likelihood (6) has a nice form of a multiplication of the normal density functions and allows us to develop a computationally efficient Bayesian estimation approach.

### 3.2 Review of several Bayesian variable selection priors

Since this article considers the case that there are many potential covariates for both types of responses, we would like to develop Bayesian variable selection techniques in this framework by adopting different priors for the two sets of regression parameters. Here we provide a brief review of several Bayesian variable selection priors for regression parameters in the conventional linear model setting. The linear model specifies

$$y=1_{n}\mu +X\beta +\epsilon$$

where y is an n×1 vector of responses, $1_{n}$ is an n×1 vector with all elements being 1, X is an n×p matrix of regressors, and ϵ is an n×1 vector of independent and identically distributed normal errors with mean 0 and variance $\sigma^{2}$.

The Lasso estimator of the regression parameters β can be viewed as the minimizers of the following penalized least squares with $L_{1}$ penalty (Tibshirani, 1996),

$${\left(y-1_{n}\mu -X\beta \right)}^{T}\left(y-1_{n}\mu -X\beta \right)+\lambda \sum_{j=1}^{p}\left|\beta_{j}\right|,$$

where λ is a positive parameter that controls the degree of penalty. This Lasso estimator can also be viewed as the posterior mode in a Bayesian framework when a double-exponential prior is assigned for the regression parameters β. Park and Casella (2008) proposed Bayesian Lasso using a conditional Laplace prior specification given $\sigma^{2}$

$$\pi \left(\beta |\sigma^{2}\right)=\prod_{j=1}^{p}\frac{\lambda }{2\sqrt{\sigma^{2}}}\text{exp}\left(-\frac{\lambda \left|\beta_{j}\right|}{\sqrt{\sigma^{2}}}\right)$$

and developed a Gibbs sampler for the Bayesian Lasso. Their Gibbs sampler exploited the representation of the Laplace distribution as a scale mixture of normals with an exponential mixing density (Andrews and Mallows, 1974),

$$\frac{a}{2}e^{-a|z|}=\int_{0}^{\infty }\frac{1}{\sqrt{2\pi s}}\text{exp}\left(-\frac{z^{2}}{2s}\right)\frac{a^{2}}{2}\text{exp}\left(-\frac{a^{2}s}{2}\right)ds,\;a>0.$$

This eventually leads to the following hierarchical representation of the Bayesian Lasso,

$$\beta_{j}|\sigma^{2},\tau_{j}^{2}\overset{ind}{\sim }N\left(0,\sigma^{2}\tau_{j}^{2}\right),\;j=1,\ldots ,p,\;\tau_{j}^{2}\overset{iid}{\sim }ℰ\left(\lambda^{2}/2\right),\;j=1,\ldots ,p,\;\left(\sigma^{2},\lambda \right)\sim \pi \left(\sigma^{2}\right)\times \pi \left(\lambda \right),$$

where ℰ(a) denotes an Exponential distribution with rate parameter a.

Bayesian adaptive Lasso is a generalization of Bayesian Lasso by allowing different penalties to be used for different regression parameters (Zou, 2006), and specifically, the adaptive Lasso estimator is the minimizer of the following penalized function

$${\left(y-1_{n}\mu -X\beta \right)}^{T}\left(y-1_{n}\mu -X\beta \right)+\sum_{j=1}^{p}\lambda_{j}\left|\beta_{j}\right|,$$

where $\lambda_{j}$ is a positive penalty parameter associated with $\beta_{j}$ for j=1,…,p. The hierarchical structure of the Bayesian adaptive Lasso prior (Leng et al., 2014) is

$$\beta_{j}|\sigma^{2},\tau_{j}^{2}\overset{ind}{\sim }N\left(0,\sigma^{2}\tau_{j}^{2}\right),\;j=1,\ldots ,p,\;\tau_{j}^{2}\overset{ind}{\sim }ℰ\left(\lambda_{j}^{2}/2\right),\;j=1,\ldots ,p,\;\left(\sigma^{2},\lambda_{1},\cdots ,\lambda_{p}\right)\sim \pi \left(\sigma^{2}\right)\times \prod_{j=1}^{p}\pi \left(\lambda_{j}\right).$$

The spike-and-slab prior has also been widely used for variable selection (Mitchell and Beauchamp, 1988; George and McCulloch, 1997; Ishwaran and Rao, 2005). The spike component concentrates its mass near zero, and allows to shrink small effects towards zero. On the other hand, the slab component has its mass spread over a wide range of plausible values and can help identify nonzero parameters.

The spike-and-slab prior is usually accompanied with a stochastic search variable selection process. First, indicator variables $\Delta =\left(\Delta_{1},\ldots ,\Delta_{p}\right)$ are used to indicate the allocation of a regression coefficient to the slab component or the spike component. Here we define $\Delta_{j}=1$ if $\beta_{j}$ is from the slab component. The variance of the normal spike prior $\sigma_{\text{spike}}^{2}$ is taken to be much smaller than that of the normal slab prior variance $\sigma_{\text{slab}}^{2}$. The allocation probability $P\left(\Delta_{j}=1\right)=\omega_{j}$ is specified to follow a Beta distribution with shape parameters $a_{\omega }$ and $b_{\omega }$. Accordingly, the spike-and-slab prior has the following hierarchical structure

$$\beta_{j}|\Delta_{j}=1\sim N\left(0,\sigma_{\text{slab }}^{2}\right),\;\beta_{j}|\Delta_{j}=0\sim N\left(0,\sigma_{\text{spike }}^{2}\right),\;\Delta_{j}\sim \text{Ber}\left(\omega_{j}\right),\;\omega_{j}\sim ℬ\left(a_{\omega },b_{\omega }\right),$$

where Ber and ℬ denote the Bernoulli and Beta distributions, respectively.

### 3.3 Bayesian variable selection priors for regression parameters

Now we apply the Bayesian Lasso priors, Bayesian adaptive Lasso priors, and the spike-and-slab priors to both sets of regression parameters β and θ under the proposed models for longitudinal and interval-censored data. Specifically, the Bayesian Lasso priors for β and θ have the following hierarchical structures

$$\beta |\tau_{s,1}^{2},\cdots ,\tau_{s,p}^{2}\sim N_{p}\left(0_{p},D_{\tau_{s}}\right),\;D_{\tau_{s}}=\text{diag}\left(\tau_{s,1}^{2},\cdots ,\tau_{s,p}^{2}\right),\;\tau_{s,j}^{2}\sim ℰ\left(\lambda_{s}^{2}/2\right),\;j=1,\ldots ,p,\;\lambda_{s}\sim 𝒢a\left(a_{\lambda },b_{\lambda }\right).\;\theta |\sigma_{\epsilon }^{2},\tau_{l,1}^{2},\cdots ,\tau_{l,p}^{2}\sim N_{p}\left(0_{p},\sigma_{\epsilon }^{2}D_{\tau_{l}}\right),\;D_{\tau_{l}}=\text{diag}\left(\tau_{l,1}^{2},\cdots ,\tau_{l,p}^{2}\right),\;\tau_{l,j}^{2}\sim ℰ\left(\lambda_{l}^{2}/2\right),\;j=1,\ldots ,p,\;\lambda_{l}\sim 𝒢a\left(a_{\lambda },b_{\lambda }\right),\;\sigma_{\epsilon }^{-2}\sim 𝒢a\left(a_{\epsilon },b_{\epsilon }\right),$$

where $𝒢a\left(a_{\epsilon },b_{\epsilon }\right)$ denotes the gamma distribution with shape parameter $a_{\epsilon }$ and rate parameter $b_{\epsilon }$.

The Bayesian adaptive Lasso priors for β and θ have the following hierarchical structures,

$$\beta |\tau_{s,1}^{2},\cdots ,\tau_{s,p}^{2}\sim N_{p}\left(0_{p},\;D_{\tau_{s}}\right),\;D_{\tau_{s}}=\text{diag}\left(\tau_{s,1}^{2},\cdots ,\tau_{s,p}^{2}\right),\;\tau_{s,j}^{2}\sim ℰ\left(\lambda_{s,j}^{2}/2\right),\;\lambda_{s,j}\sim 𝒢a\left(a_{\lambda },b_{\lambda }\right),\;j=1,\ldots ,p,\;\theta |\sigma_{\epsilon }^{2},\tau_{l,1}^{2},\cdots ,\tau_{l,p}^{2}\sim N_{p}\left(0_{p},\sigma_{\epsilon }^{2}D_{\tau_{l}}\right),\;D_{\tau_{l}}=\text{diag}\left(\tau_{l,1}^{2},\cdots ,\tau_{l,p}^{2}\right),\;\tau_{l,j}^{2}\sim ℰ\left(\lambda_{l,j}^{2}/2\right),\;\lambda_{l,j},\sim 𝒢a\left(a_{\lambda },b_{\lambda }\right),\;j=1,\ldots ,p,\;\sigma_{\epsilon }^{-2}\sim 𝒢a\left(a_{\epsilon },b_{\epsilon }\right).$$

The spike-and-slab priors for β and θ have the following hierarchical structure

$$\beta_{j}|\Delta_{s,j}=1\sim N\left(0,\sigma_{\text{slab }}^{2}\right),\;\beta_{j}|\Delta_{s,j}=0\sim N\left(0,\sigma_{\text{spike }}^{2}\right),\;\Delta_{s,j}\sim \text{Ber}\left(\omega_{s,j}\right),\;\omega_{s,j}\sim ℬ\left(a_{\omega },b_{\omega }\right);\;\theta_{j}|\Delta_{l,j}=1\sim N\left(0,\sigma_{\epsilon }^{2}\sigma_{\text{slab }}^{2}\right),\;\theta_{j}|\Delta_{l,j}=0\sim N\left(0,\sigma_{\epsilon }^{2}\sigma_{\text{spike }}^{2}\right),\;\Delta_{l,j}\sim \text{Ber}\left(\omega_{l,j}\right),\;\omega_{l,j}\sim ℬ\left(a_{\omega },b_{\omega }\right).$$

For the purpose of comparison, we also consider a basic model using a general multivariate normal prior for β and θ

$$\beta \sim N_{p}\left(\beta_{0},\Sigma_{\beta }\right)\text{ and }\theta \sim N_{p}\left(\theta_{0},\Sigma_{\theta }\right).$$

### 3.4 Prior specifications for other parameters

In addition to the above prior specifications for the regression parameters β and θ, we assign a Gamma prior $𝒢a\left(a_{u},b_{u}\right)$ for $\sigma_{u}^{-2}$, a Gamma prior $𝒢a\left(a_{\xi },b_{\xi }\right)$ for $\sigma_{\xi }^{-2}$, a multivariate normal prior $N_{\tilde{K}}\left(\eta_{0},\Sigma_{\eta }\right)$ for η, a normal prior $N\left(m_{0},\nu_{0}^{-1}\right)$ for $r_{0}$, and independent exponential priors Exp⁡(ρ) for ${\left\{r_{k}\right\}}_{k=1}^{K^{*}}$. The adoption of independent exponential priors for ${\left\{r_{k}\right\}}^{\prime }s$ serves to shrink small coefficients to zero and helps prevent over-fitting problems due to the use of many knots in the monotone spline specification (Lin and Wang, 2010; Wang and Dunson, 2011). To allow the data to inform the degree of shrinkage, we treat ρ as random and assign a Gamma prior $𝒢a\left(a_{\rho },b_{\rho }\right)$ for ρ.

## 4 Proposed Gibbs samplers

Gibbs samplers can be derived based on the complete data likelihood (6) and the priors specified in Sections 3.3 and 3.4. Below we present the Gibbs sampler associated with the Bayesian Lasso priors and refer it as Bayesian Lasso Gibbs sampler. The proposed Gibbs samplers developed based on other prior specifications in Section 3.3 are similar to the Bayesian Lasso Gibbs sampler but with some modifications.

1. For i=1,…,n, sample $z_{i}$ from a truncated normal distribution
$$N\left(\alpha \left(t_{i}\right)+X_{i}^{\prime }\beta +u_{i},1\right)1_{C_{i}}\left(z_{i}\right),$$
  where $C_{i}$ takes the form of (0,∞) if $\delta_{i1}=1,\left(\alpha \left(L_{i}\right)-\alpha \left(R_{i}\right),0\right)$ if $\delta_{i2}=1$, and (-∞,0) if $\delta_{i3}=1$ for subject i.
2. Sample $r_{0}$ from $N\left(E_{0},W_{0}^{-1}\right)$, where $W_{0}=v_{0}+n$ and
$$E_{0}=W_{0}^{-1}\left[v_{0}m_{0}+\sum_{i=1}^{n}\left\{z_{i}-\sum_{k=1}^{K}r_{k}b_{k}\left(t_{i}\right)-X_{i}^{\prime }\beta -u_{i}\right\}\right].$$
3. Sample $r_{k}$ for $k=1,\ldots ,K^{*}$. Let $W_{k}=\sum_{i=1}^{n}b_{k}^{2}\left(t_{i}\right)$ for $k=1,\ldots ,K^{*}$. Sample $r_{k}$ from the prior Exp⁡(ρ) if $W_{k}=0$; otherwise, sample $r_{k}$ from a truncated normal distribution $N\left(E_{k},W_{k}^{-1}\right)1_{\left(r_{k}>d_{k}^{*}\right)}$, where
$$E_{k}=W_{k}^{-1}\left[\sum_{i=1}^{n}b_{k}\left(t_{i}\right)\left\{z_{i}-r_{0}-\sum_{k^{\prime }\neq k}r_{k^{\prime }}b_{k^{\prime }}\left(t_{i}\right)-X_{i}^{\prime }\beta -u_{i}\right\}-\rho \right],\;d_{k}^{*}=\text{max}\left(c_{k}^{*},0\right),\text{ and }c_{k}^{*}=\underset{i:\delta_{i2}=1}{\text{max}}\left[\frac{-z_{i}-\sum_{k^{\prime }\neq k}\left\{b_{k^{\prime }}\left(R_{i}\right)-b_{k^{\prime }}\left(L_{i}\right)\right\}}{b_{k}\left(R_{i}\right)-b_{k}\left(L_{i}\right)}\right].$$
4. Sample ρ from $𝒢a\left(a_{\rho }+K^{*},b_{\rho }+\sum_{k=1}^{K^{*}}r_{k}\right)$.
5. Sample β from $N_{p}\left({\tilde{\Sigma }}_{\beta }\sum_{i=1}^{n}\left\{z_{i}-\alpha \left(t_{i}\right)-u_{i}\right\}X_{i},\;{\tilde{\Sigma }}_{\beta }\right)$, where ${\tilde{\Sigma }}_{\beta }=\left(D_{\tau_{s}}^{-1}+\right.{\left.\sum_{i=1}^{n}X_{i}X_{i}^{\prime }\right)}^{-1}$ and $D_{\tau_{s}}=\text{diag}\left(\tau_{s,1}^{2},\cdots ,\tau_{s,p}^{2}\right)$.
6. Sample $\lambda_{s}$ from $𝒢a\left(p+a_{\lambda },\frac{1}{2}\sum_{j=1}^{p}\tau_{s,j}^{2}+b_{\lambda }\right)$.
7. Sample $\tau_{s,j}^{-2}$ from an inverse Gaussian distribution $IG\left({\left(\lambda_{s}^{2}/\beta_{j}^{2}\right)}^{\frac{1}{2}},\lambda_{s}^{2}\right)$ with mean parameter ${\left(\lambda_{s}^{2}/\beta_{j}^{2}\right)}^{\frac{1}{2}}$ and scale parameter $\lambda_{s}^{2}$ for j=1,…,p.
8. Sample θ from $N_{p}\left(\tilde{\theta },{\tilde{\Sigma }}_{\theta }\right)$, where ${\tilde{\Sigma }}_{\theta }=\sigma_{\epsilon }^{2}{\left(D_{\tau_{l}}^{-1}+\sum_{i=1}^{n}m_{i}X_{i}X_{i}^{\prime }\right)}^{-1},D_{\tau_{l}}=\text{diag}\left(\tau_{l,1}^{2},\cdots ,\tau_{l,p}^{2}\right)$, and
$$\tilde{\theta }={\left(D_{\tau_{l}}^{-1}+\sum_{i=1}^{n}m_{i}X_{i}X_{i}^{\prime }\right)}^{-1}\sum_{i=1}^{n}\sum_{j=1}^{m_{i}}\left\{y_{ij}-\mu \left(t_{ij}\right)-u_{i}-\xi_{i}\right\}X_{i}.$$
9. Sample $\lambda_{l}$ from $𝒢a\left(p+a_{\lambda },\frac{1}{2}\sum_{j=1}^{p}\tau_{l,j}^{2}+b_{\lambda }\right).$
10. Sample $\tau_{l,j}^{-2}$ from $IG\left({\left(\lambda_{l}^{2}\sigma_{\epsilon }^{2}/\theta_{j}^{2}\right)}^{\frac{1}{2}},\lambda_{l}^{2}\right)$ for j=1,…,p.
11. Sample η from $N_{\tilde{K}}\left(\tilde{\eta },{\tilde{\Sigma }}_{\eta }\right)$, where ${\tilde{\Sigma }}_{\eta }={\left(\Sigma_{\eta }^{-1}+\sigma_{\epsilon }^{-2}A\right)}^{-1},A$ is a matrix with its $\left(h,h^{\prime }\right)$ th element being $\sum_{i=1}^{n}\sum_{j=1}^{m_{i}}{\tilde{M}}_{h}\left(t_{ij}\right){\tilde{M}}_{h^{\prime }}\left(t_{ij}\right)$ for $1\leq h,h^{\prime }\leq \tilde{K}$, and
$$\tilde{\eta }={\tilde{\Sigma }}_{\eta }\left\{\Sigma_{\eta }^{-1}\eta_{0}+\sigma_{\epsilon }^{-2}\sum_{i=1}^{n}\sum_{j=1}^{m_{i}}\left(y_{ij}-X_{i}^{\prime }\theta -u_{i}-\xi_{i}\right)\tilde{M}\left(t_{ij}\right)\right\}.$$
12. Sample $u_{i}$ from
$$N\left(V_{i}^{-1}\left[\sigma_{\epsilon }^{-2}\sum_{j=1}^{m_{i}}\left\{y_{ij}-\mu \left(t_{ij}\right)-X_{i}^{\prime }\theta -\xi_{i}\right\}+\left\{z_{i}-\alpha \left(t_{i}\right)-X_{i}^{\prime }\beta \right\}\right],V_{i}^{-1}\right).$$
  where $V_{i}=m_{i}\sigma_{\epsilon }^{-2}+\sigma_{u}^{-2}+1$ for i=1,…,n.
13. Sample $\sigma_{u}^{-2}$ from $𝒢a\left(\frac{1}{2}n+a_{u},\frac{1}{2}\sum_{i=1}^{n}u_{i}^{2}+b_{u}\right)$.
14. Sample $\xi_{i}$ from
$$N\left({\left(m_{i}\sigma_{\epsilon }^{-2}+\sigma_{\xi }^{-2}\right)}^{-1}\left[\sigma_{\epsilon }^{-2}\sum_{j=1}^{m_{i}}\left\{y_{ij}-\mu \left(t_{ij}\right)-X_{i}^{\prime }\theta -u_{i}\right\}\right],{\left(m_{i}\sigma_{\epsilon }^{-2}+\sigma_{\xi }^{-2}\right)}^{-1}\right).$$
15. Sample $\sigma_{\xi }^{-2}$ from $𝒢a\left(\frac{n}{2}+a_{\xi },\frac{1}{2}\sum_{i=1}^{n}\xi_{i}^{2}+b_{\xi }\right)$.
16. Sample $\sigma_{\epsilon }^{-2}$ from
$$𝒢a\left(a_{\epsilon }+\frac{1}{2}\sum_{i=1}^{n}m_{i}+\frac{p}{2},b_{\epsilon }+\frac{1}{2}\sum_{i=1}^{n}\sum_{j=1}^{m_{i}}{\left\{y_{ij}-\mu \left(t_{ij}\right)-X_{i}^{\prime }\theta -u_{i}-\xi_{i}\right\}}^{2}+\frac{1}{2}\theta^{\prime }D_{\tau_{l}}^{-1}\theta \right).$$

The proposed Gibbs sampler based on the Bayesian adaptive Lasso prior in Section 3.3 modifies the above Bayesian Lasso Gibbs sampler by replacing Steps 6, 7, 9, and 10 with the following steps.

6a. Sample $\lambda_{s,j}$ from $𝒢a\left(1+a_{\lambda },\frac{1}{2}\tau_{s,j}^{2}+b_{\lambda }\right)$ for j=1,…,p.
7a. Sample $\tau_{s,j}^{-2}$ from $IG\left({\left(\lambda_{s,j}^{2}/\beta_{j}^{2}\right)}^{\frac{1}{2}},\lambda_{s,j}^{2}\right)$ for j=1,…,p.
9a. Sample $\lambda_{l,j}$ from $𝒢a\left(1+a_{\lambda },\frac{1}{2}\tau_{l,j}^{2}+b_{\lambda }\right)$ for j=1,…,p.
10a. Sample $\tau_{l,j}^{-2}$ from $IG\left({\left(\lambda_{l,j}^{2}\sigma_{\epsilon }^{2}/\theta_{j}^{2}\right)}^{\frac{1}{2}},\lambda_{l,j}^{2}\right)$ for j=1,…,p.

To accommodate the spike-and-slab prior, one needs to sample the indicator variables $\Delta_{s,j}$’s and $\Delta_{l,j}$’s, the inclusion probabilities $\omega_{s}$’s and $\omega_{l}$’s, and regression coefficients β and θ in the posterior computation. The proposed spike-and-slab Gibbs sampler modifies the Bayesian Lasso Gibbs sampler by replacing its Steps 5–10 with the following steps.

5b. For j=1,…,p, sample $\Delta_{s,j}$ from $\text{Ber}\left({\left\{1+R_{s,j}\left(1-\omega_{s}\right)/\omega_{s}\right\}}^{-1}\right)$, where
$$R_{s,j}=\text{exp}\left\{-0.5\beta_{j}^{2}\left(\sigma_{\text{spike }}^{-2}-\sigma_{\text{slab }}^{-2}\right)\right\}\sigma_{\text{slab }}/\sigma_{\text{spike }}$$
6b. Sample $\omega_{s}$ from $ℬ\left(a_{\omega }+\sum_{j=1}^{p}\Delta_{s,j},b_{\omega }+p-\sum_{j=1}^{p}\Delta_{s,j}\right)$.
7b. Sample β from $N_{p}\left({\tilde{\Sigma }}_{\beta }\sum_{i=1}^{n}\left\{z_{i}-\alpha \left(t_{i}\right)-u_{i}\right\}X_{i},{\tilde{\Sigma }}_{\beta }\right)$, where ${\tilde{\Sigma }}_{\beta }=\left(D_{\sigma \beta }^{-1}+\right.{\left.\sum_{i=1}^{n}X_{i}X_{i}^{\prime }\right)}^{-1},D_{\sigma \beta }=\text{diag}\left(\sigma_{\beta_{1}|\Delta_{s,1}^{2}}^{2},\cdots ,\sigma_{\beta_{p}|\Delta_{s,p}}^{2}\right)$, and $\sigma_{\beta_{j}|\Delta_{s,j}}^{2}$ takes $\sigma_{\text{slab}}^{2}$ if $\Delta_{s,j}=1$ and $\sigma_{\text{spike}}^{2}$ if $\Delta_{s,j}=0$ for j=1,…,p.
8b. For j=1,…,p, sample $\Delta_{l,j}$ from $\text{Ber}\left({\left\{1+R_{l,j}\left(1-\omega_{l}\right)/\omega_{l}\right\}}^{-1}\right)$, where
$$R_{l,j}=\text{exp}\left\{-0.5\left(\theta_{j}^{2}/\sigma_{\epsilon }^{2}\right)\left(\sigma_{\text{spike }}^{-2}-\sigma_{\text{slab }}^{-2}\right)\right\}\sigma_{\text{slab }}/\sigma_{\text{spike }}.$$
9b. Sample $\omega_{l}$ from $ℬ\left(a_{\omega }+\Sigma_{j=1}^{p}\Delta_{l,j},b_{\omega }+p-\Sigma_{j=1}^{p}\Delta_{l,j}\right)$.
10b. Sample θ from $N_{p}\left(\tilde{\theta },{\tilde{\Sigma }}_{\theta }\right)$, where ${\tilde{\Sigma }}_{\theta }=\sigma_{\epsilon }^{2}{\left(D_{\sigma \theta }^{-1}+\sum_{i=1}^{n}m_{i}X_{i}X_{i}^{\prime }\right)}^{-1},\tilde{\theta }={\left(D_{\sigma \theta }^{-1}+\sum_{i=1}^{n}m_{i}X_{i}X_{i}^{\prime }\right)}^{-1}\sum_{i=1}^{n}\sum_{j=1}^{m_{i}}\left\{y_{ij}-\mu \left(t_{ij}\right)-u_{i}-\xi_{i}\right\}X_{i},\;D_{\sigma \theta }=\text{diag}\left(\sigma_{\theta_{1}|\Delta_{l,1}}^{2},\cdots ,\sigma_{\theta_{p}|\Delta_{l,p}}^{2}\right)$, and $\sigma_{\theta_{j}|\Delta_{l,j}}^{2}$ takes $\sigma_{\text{slab}}^{2}$ if $\Delta_{l,j}=1$ and $\sigma_{spike}^{2}$ if $\Delta_{l,j}=0$ for j=1,…,p.

The proposed Gibbs sampler based on the multivariate normal priors for β and θ is referred to as General Bayesian Gibbs sampler, which replaces Steps 5–10 of the Bayesian Lasso Gibbs sampler with the following two steps.

c1. Sample β from $N_{p}\left(\tilde{\beta },{\tilde{\Sigma }}_{\beta }\right)$, where ${\tilde{\Sigma }}_{\beta }={\left(\Sigma_{\beta }^{-1}+\sum_{i=1}^{n}X_{i}X_{i}^{\prime }\right)}^{-1}$ and
$$\tilde{\beta }={\tilde{\Sigma }}_{\beta }\left[\Sigma_{\beta }^{-1}\beta_{0}+\sum_{i=1}^{n}\left\{z_{i}-\alpha \left(t_{i}\right)-cu_{i}\right\}X_{i}\right].$$
c2. Sample θ from $N_{p}\left(\tilde{\theta },{\tilde{\Sigma }}_{\theta }\right)$, where ${\tilde{\Sigma }}_{\theta }={\left(\Sigma_{\theta }^{-1}+\sigma_{\epsilon }^{-2}\sum_{i=1}^{n}m_{i}X_{i}X_{i}^{\prime }\right)}^{-1}$ and
$$\tilde{\theta }={\tilde{\Sigma }}_{\theta }\left[\Sigma_{\theta }^{-1}\theta_{0}+\sigma_{\epsilon }^{-2}\sum_{i=1}^{n}\sum_{j=1}^{m_{i}}\left\{y_{ij}-\mu \left(t_{ij}\right)-u_{i}\right\}X_{i}\right].$$

It is appealing that the full conditional distributions of all the parameters and latent variables are well recognized distributions with explicit forms in each of the proposed Gibbs samplers. Thus, sampling these unknowns are straightforward, and no black-box or any complicated Metropolis steps are required. These properties make proposed Gibbs samplers easy to implement and computationally efficient.

## 5 Simulation studies

Simulation studies were conducted to evaluate the performance of our proposed methods. The observed longitudinal and survival data were generated as follows. First we generated the observation process. For subject i, we generated the number of observations $m_{i}$ from a Poisson distribution with mean 3 plus 1 but with a cap of 10. Then we generated $m_{i}$ gap times independently from an exponential distribution with mean 0.5 and obtained the observation times $t_{ij}$’s as the cumulative sums of these gap times. Next we generated the longitudinal responses and the survival time from models (1) and (2), respectively. To specify the models (1) and (2), we set $\mu \left(t\right)=4\text{log}\left(t+1\right)-2t,\alpha (t)=2\text{log}(t)+t^{2},\sigma_{\xi }^{2}=0.25,\sigma_{u}^{2}=0.25$ or 0.5, and $\sigma_{\epsilon }^{2}=0.25$ or 1. The following two scenarios were considered for regression parameters (θ,β) to evaluate the proposed methods on variable selection,

- $\theta =(1,0,1,0,1,0,1,0,1,0{)}^{\prime }$, and $\beta =(1,1,0,0,1,1,0,0,1,0{)}^{\prime }$.
- $\theta ={\left(1_{10},0_{20}\right)}^{\prime }$, and $\beta ={\left(1_{5},0_{5},1_{5},0_{15}\right)}^{\prime }$.

Here $0_{n}$ and $1_{n}$ are n dimensional vectors with all values equal to zero and one, respectively. These two scenarios represent regular and sparse cases of important covariates with a total number of covariates p=10 and 30, respectively. Equal number of covariates were generated from N(0,1) and Bernoulli (0.5) for both scenarios. With all these specifications, the longitudinal responses and the failure times were then generated from the longitudinal model (1) and the survival model (2), respectively. The observed interval for the failure time of each subject was determined by the two adjacent observation times (i.e., $t_{ij}$’s together with 0 and ∞) that contained the failure time. For each parameter setting, 500 data sets were generated, each with a sample size n=500.

For the spline specifications in modeling μ(⋅) and α(⋅), we chose cubic basis functions (i.e $\tilde{d}=d^{*}=3$) and used 3 interior knots for each set of M-splines, i.e., $\tilde{J}=J^{*}=3$. The interior knots were specified as the quantiles of the observation times $t_{ij}$’s for modeling μ and the quantiles of the end points of the observed intervals ($\left.L_{i},R_{i}\right]$’s excluding 0 and ∞ for modeling α. These specifications result in 7 basis functions for each set, i.e., $\tilde{K}=K^{*}=7$.

We adopted the following prior specifications for the unknown parameters, $a_{u}=b_{u}=0.1$ leading to a 𝒢a(0.1,0.1) prior for $\sigma_{u}^{-2},a_{\xi }=b_{\xi }=0.1$ leading to a 𝒢a(0.1,0.1) prior for $\sigma_{\xi }^{-2},m_{0}=-6$ and $v_{0}=1$ leading to a N(-6,1) prior for $r_{0},a_{\rho }=b_{\rho }=1$ leading to a 𝒢a(1,1) prior for ρ, a multivariate normal prior $N_{p}\left(\eta_{0}=0_{\tilde{k}},\Sigma_{\eta }=100I_{\tilde{K}}\right)$ for $\eta ={\left(\eta_{1},\ldots \eta_{\tilde{k}}\right)}^{\prime },a_{\epsilon }=b_{\epsilon }=0.1$ leading to a 𝒢a(0.1,0.1) prior for $\sigma_{\epsilon }^{-2},a_{\lambda }=1$ and $b_{\lambda }=2$ leading to a 𝒢a(1,2) prior for all the Lasso parameters, and $a_{\omega }=b_{\omega }=1$ leading to a B(1,1) prior for all the allocation probabilities. The $\sigma_{\text{spike}}^{2}$ and $\sigma_{\text{slab}}^{2}$ were taken to be 0.01 and 100, respectively. We also used a multivariate normal prior $N_{p}\left(0_{p},100I_{p}\right)$ for both β and θ in the more basic model.

We ran each of the proposed Gibbs samplers with a chain of 5,000 iterations, and fast convergence of the MCMC was observed for all of our Gibbs samplers in the simulation. Statistical summaries such as the posterior means and quantiles were obtained based on the 4,000 iterations of MCMC after discarding the first 1,000 iterations as a burn-in for each Gibbs sampler and for each data set.

Tables 1 and 2 display BIAS, the difference between the average of the 500 posterior means and the true value, RMSE, the root mean squared errors based on the 500 point estimates, and CP95, the coverage probability of 95% credible intervals, for parameters based on the proposed Bayesian approaches across the settings in terms of $\sigma_{u}^{2}$ and $\sigma_{\epsilon }^{2}$. As seen in the two tables, all of our proposed methods work well with small bias and the 95% coverage probabilities being close to the nominal value 0.95 for all parameters. The Bayesian Lasso, Bayesian adaptive Lasso, and spike-and-slab methods produce smaller RMSEs for most of the parameters across the setups than the general Bayesian method, especially for the parameters of the unimportant covariates (i.e, those with their corresponding coefficients equal to 0). Among these four methods, the spike-and-slab method has the smallest RMSEs for the parameters of the unimportant covariates.

We also compare the performance of these four Bayesian methods from variable selection point of view. For each method, a regression parameter is labeled as “negative” if its 95% credible interval contains 0 and as “positive” otherwise. Tables 3 and 4 present the results of variable selection accuracy in terms of average size (aver.size): average number of regression parameters labelled as “positive”; true negatives (TN): average number of regression parameters labelled as “negative” correctly; and false negatives (FN): average number of regression parameters labelled as “negative” incorrectly. The true average size and true negative are 5 and 5, respectively in Scenario I and are 10 and 20, respectively in Scenario II. Several conclusions can be drawn from the two tables. First, all the methods have similar variable selection performance across the parameter settings in terms of $\sigma_{u}^{2}$ and $\sigma_{\epsilon }^{2}$. Second, the three variable selection methods (BL, BAL, and SS) perform better than GB in general with average size and number of true negatives closer to the true values. Third, SS performs best among all methods in Scenario I but performs worst in the survival submodel in Scenario II. Overall, the three variable section methods are comparable based on our simulation study.

## 6 Real-life data application

Initiated in the 1970s, the Aerobic Center Longitudinal Study (ACLS) aimed to investigate health outcomes associated with physical activity and cardiorespiratory fitness (Sui et al., 2013) at the Cooper Clinic in Dallas, Texas. Patients visited the private clinic periodically and received extensive medical examinations during each visit. More information about this study can be found in Sui et al. (2007) and Sui et al. (2017).

Here we consider the ACLS data from the 13,827 patients who had at least two clinic visits recorded between 1974 and 2006 and focus on a joint analysis of blood cholesterol level and the time to hypertension. The total cholesterol levels were measured in millimoles per litre (mmol/L) in this study, and hypertension was defined as a systolic blood pressure of 140 mmHg or higher, a diastolic blood pressure of 90 mmHg or higher, or physician diagnosis of hypertension (Sui et al., 2017). Cholesterol level was measured for all patients at each visit and thus has a longitudinal data structure. Blood presure was also measured at each visit, and the time to hypertension has a structure of interval-censored data. The left, interval, and right censoring rates for the time to hypertension are 29.1%, 24.0%, and 46.9%, respectively.

Twenty-five covariates are considered in our analysis, and we are interested in variable selection and parameter estimation in this joint analysis. These covariates include age and body mass index (BMI) at entry, gender (1 for female and 0 for male), parental history of high blood pressure, diabetes, and premature coronary heart disease, depression, anxiety, smoking status, and heavy drinking (1 for yes and 0 otherwise), marital status (1 for being married and 0 otherwise), and physical activity status (1 for being inactive and 0 otherwise). The two continuous covariates are standardized before fitting the model. For the splines specifications, we choose degree to be 3 and take 3 interior knots placed at the quartiles for both sets of M-splines. Bayesian Lasso, Bayesian adaptive Lasso, spike-and-slab, and the general Bayesian methods proposed in Section 4 are implemented for the variable selection in the context of joint modeling. The same prior specifications are adopted as in the simulation study in Section 5. A total of 15,000 iterations were run for each of our Gibbs samplers, and the first 5,000 iterations were discarded as a burn-in. Statistical summaries were calculated based on the latter 10,000 iterations of the chains for each Gibbs sampler.

Tables 5 and 6 present the estimated covariate effects as well as their 95% credible intervals on the longitudinal and survival responses, respectively. As seen in these two tables, the four methods yield very similar results, although the general Bayesian method seems to give slightly wider 95% credible intervals than the three other methods. For the longitudinal analysis, females in this study were more likely to have higher levels of cholesterol than their male counterparts. Also, patients who reported heavy drinking, chest pain or pressure, no leisure-time physical activity, being currently unmarried and parental history of coronary heart disease, whose initial triglyceride was higher, and who were enrolled at an older age were more likely to have higher levels of cholesterol. For the survival analysis, those who were males, had higher levels of initial BMI, reported chest pain or pressure, rapid or irregular heartbeats, heavy drinking, being currently unmarried, no leisure-time physical activity, and parental history of high blood pressure and of premature coronary heart disease, and were enrolled at a younger age were more likely to develop hypertension earlier in their life.

Other specifications of the monotone splines in the proposed methods were also investigated. Table 7 summarizes the estimation results of the variance parameters and Kendall’s τ when using different numbers of interior knots. As seen clearly in Table 7, the estimation results are very close across the numbers of interior knots for all the proposed methods. This indicates that the proposed methods are robust to the monotone spline specifications, and this phenomenon was also observed in other works on monotone splines in literature (Ramsay, 1988; Wang and Dunson, 2011; Wang et al., 2016).

## 7 Discussion

Although Bayesian Lasso method developed by Park and Casella (2008) has been used in various models such as linear regression models (Hans, 2009; Lykou and Ntzoufras, 2013) and semiparametric structural equation models (Guo et al., 2012), there is a general dearth of work on it in the context of joint analysis of longitudinal and survival data. In this article, we develop several Bayesian variable selection methods including Bayesian Lasso in joint analysis of longitudinal and interval-censored data. The proposed Gibbs samplers are computationally efficient because all of the full conditionals are standard distributions and are easy to sample from, based on our well calibrated data augmentation techniques. The proposed methods are shown to have excellent estimation performance as well as good variable selection performance in our simulation study.

The proposed methods can be extended to more advanced models. For example, one may incorporate more random effects terms or time-varying covariates in the longitudinal submodel. One may also relax the normal frailty assumption in the joint model by allowing a nonparameteric distribution for the shared frailty. Future research will be devoted to the exploration of such extensions.

## Figures and Tables

**Table 1:** Summary statistics for the simulation studies considering 10 covariates when $\left(\sigma_{u}^{2},\sigma_{\epsilon }^{2}\right)=(0.25,1)$ and (0.25,0.25). True $\theta =(1,0,1,0,1,0,1,0,1,0{)}^{\prime },\beta =(1,1,0,0,1,1,0,0,1,0{)}^{\prime }$, and $\sigma_{\xi }^{2}=0.25$.

		BL			BAL			SS			GB		
$\left(\sigma_{u}^{2},\sigma_{\epsilon }^{2}\right)$		BIAS	RMSE	CP95	BIAS	RMSE	CP95	BIAS	RMSE	CP95	BIAS	RMSE	CP95
(0.25, 1)	$\theta_{1}$	−0.006	0.083	0.940	−0.002	0.083	0.934	−0.012	0.083	0.936	0.004	0.083	0.934
	$\theta_{2}$	−0.003	0.071	0.970	−0.002	0.072	0.964	−0.003	0.056	0.986	−0.001	0.075	0.960
	$\theta_{3}$	−0.016	0.083	0.930	−0.013	0.083	0.930	−0.021	0.083	0.930	−0.009	0.082	0.932
	$\theta_{4}$	−0.002	0.078	0.946	−0.002	0.080	0.938	−0.001	0.061	0.972	−0.002	0.083	0.926
	$\theta_{5}$	−0.013	0.080	0.954	−0.009	0.080	0.948	−0.019	0.081	0.944	−0.003	0.079	0.950
	$\theta_{6}$	−0.001	0.037	0.952	−0.001	0.038	0.956	−0.001	0.034	0.966	0.000	0.038	0.956
	$\theta_{7}$	−0.003	0.039	0.962	−0.002	0.039	0.970	−0.004	0.039	0.966	−0.001	0.039	0.970
	$\theta_{8}$	−0.002	0.038	0.962	0.002	0.039	0.960	0.001	0.035	0.968	0.002	0.039	0.968
	$\theta_{9}$	−0.002	0.040	0.946	−0.001	0.040	0.950	−0.004	0.040	0.948	0.000	0.040	0.938
	$\theta_{10}$	0.001	0.041	0.936	0.001	0.042	0.940	0.001	0.038	0.944	0.001	0.042	0.936
	$\beta_{1}$	−0.040	0.172	0.942	−0.021	0.170	0.950	−0.079	0.178	0.920	0.011	0.172	0.942
	$\beta_{2}$	−0.032	0.169	0.942	−0.014	0.169	0.950	−0.069	0.173	0.936	0.016	0.171	0.938
	$\beta_{3}$	−0.013	0.147	0.952	−0.014	0.153	0.956	−0.010	0.110	0.986	−0.014	0.167	0.936
	$\beta_{4}$	−0.005	0.147	0.958	−0.006	0.153	0.952	−0.003	0.109	0.984	−0.007	0.168	0.930
	$\beta_{5}$	−0.024	0.169	0.944	−0.004	0.169	0.952	−0.063	0.172	0.928	0.027	0.172	0.944
	$\beta_{6}$	0.007	0.099	0.948	0.018	0.101	0.942	−0.018	0.099	0.954	0.036	0.109	0.930
	$\beta_{7}$	−0.007	0.074	0.966	−0.007	0.076	0.960	−0.006	0.059	0.986	−0.007	0.081	0.952
	$\beta_{8}$	−0.005	0.078	0.942	−0.005	0.080	0.938	−0.004	0.062	0.968	−0.005	0.084	0.934
	$\beta_{9}$	0.002	0.097	0.952	0.013	0.098	0.946	−0.024	0.097	0.944	0.031	0.104	0.938
	$\beta_{10}$	0.000	0.077	0.946	0.000	0.079	0.944	0.000	0.062	0.966	0.000	0.083	0.936
	$\sigma_{u}^{2}$	−0.001	0.067	0.948	0.001	0.067	0.942	−0.011	0.066	0.944	0.008	0.068	0.942
	$\sigma_{\xi }^{2}$	0.009	0.071	0.942	0.007	0.072	0.954	0.016	0.073	0.950	0.000	0.071	0.948
	$\sigma_{\epsilon }^{2}$	0.001	0.036	0.946	0.000	0.036	0.956	0.005	0.037	0.940	0.004	0.036	0.942
(0.25, 0.25)	$\theta_{1}$	−0.012	0.072	0.930	−0.009	0.072	0.924	−0.016	0.072	0.930	−0.002	0.071	0.936
	$\theta_{2}$	−0.006	0.066	0.962	−0.005	0.066	0.958	−0.004	0.040	0.990	−0.004	0.070	0.946
	$\theta_{3}$	−0.005	0.069	0.956	−0.003	0.068	0.950	−0.007	0.068	0.946	0.002	0.069	0.942
	$\theta_{4}$	0.000	0.066	0.958	0.001	0.067	0.942	0.000	0.041	0.994	0.000	0.071	0.944
	$\theta_{5}$	−0.014	0.070	0.944	−0.011	0.070	0.944	−0.017	0.070	0.942	−0.004	0.069	0.940
	$\theta_{6}$	−0.001	0.033	0.944	−0.001	0.033	0.946	−0.001	0.025	0.976	−0.001	0.034	0.946
	$\theta_{7}$	−0.001	0.034	0.958	−0.001	0.034	0.966	−0.002	0.034	0.956	0.001	0.034	0.966
	$\theta_{8}$	−0.001	0.034	0.944	−0.001	0.034	0.946	−0.001	0.026	0.968	−0.001	0.035	0.944
	$\theta_{9}$	−0.004	0.033	0.958	−0.003	0.033	0.966	−0.005	0.033	0.958	−0.001	0.033	0.964
	$\theta_{10}$	−0.001	0.035	0.952	−0.001	0.035	0.954	0.000	0.027	0.972	0.000	0.036	0.942
	$\beta_{1}$	−0.026	0.167	0.940	−0.006	0.165	0.952	−0.062	0.170	0.928	0.025	0.171	0.944
	$\beta_{2}$	−0.035	0.175	0.932	−0.016	0.172	0.942	−0.069	0.176	0.926	0.012	0.175	0.934
	$\beta_{3}$	−0.014	0.146	0.962	−0.014	0.152	0.958	−0.011	0.111	0.978	−0.013	0.165	0.944
	$\beta_{4}$	−0.003	0.147	0.972	−0.002	0.153	0.968	−0.001	0.107	0.990	−0.003	0.168	0.954
	$\beta_{5}$	−0.032	0.176	0.920	−0.011	0.176	0.922	−0.068	0.179	0.914	0.020	0.178	0.932
	$\beta_{6}$	−0.005	0.108	0.922	0.006	0.108	0.916	−0.028	0.107	0.942	0.022	0.114	0.906
	$\beta_{7}$	−0.003	0.078	0.954	−0.003	0.080	0.948	−0.002	0.062	0.972	−0.002	0.084	0.940
	$\beta_{8}$	−0.003	0.077	0.950	−0.003	0.079	0.946	−0.002	0.062	0.974	−0.003	0.083	0.938
	$\beta_{9}$	−0.004	0.103	0.944	0.007	0.103	0.942	−0.027	0.104	0.926	0.025	0.109	0.940
	$\beta_{10}$	0.005	0.075	0.964	0.006	0.077	0.958	0.005	0.060	0.984	0.006	0.081	0.952
	$\sigma_{u}^{2}$	0.001	0.064	0.928	0.003	0.063	0.930	−0.005	0.063	0.928	0.007	0.063	0.928
	$\sigma_{\xi }^{2}$	0.004	0.064	0.934	0.002	0.062	0.942	0.009	0.063	0.938	−0.001	0.062	0.936
	$\sigma_{\epsilon }^{2}$	0.001	0.009	0.940	0.001	0.009	0.940	0.002	0.009	0.944	0.002	0.009	0.940

**Table 2:** Summary statistics for the simulation studies considering 10 covariates when $\left(\sigma_{u}^{2},\sigma_{\epsilon }^{2}\right)=(0.5,1)$ and (0.5,0.25). True $\theta =(1,0,1,0,1,0,1,0,1,0{)}^{\prime },\beta =(1,1,0,0,1,1,0,0,1,0{)}^{\prime }$, and $\sigma_{\xi }^{2}=0.25$.

		BL			BAL			SS			GB		
$\left(\sigma_{u}^{2},\sigma_{\epsilon }^{2}\right)$		BIAS	RMSE	CP95	BIAS	RMSE	CP95	BIAS	RMSE	CP95	BIAS	RMSE	CP95
(0.5, 1)	$\theta_{1}$	−0.019	0.096	0.934	−0.013	0.095	0.950	−0.029	0.097	0.930	−0.005	0.095	0.938
	$\theta_{2}$	−0.007	0.080	0.974	−0.006	0.082	0.964	−0.008	0.061	0.986	−0.003	0.085	0.956
	$\theta_{3}$	−0.005	0.092	0.938	−0.002	0.093	0.944	−0.012	0.091	0.942	0.004	0.093	0.946
	$\theta_{4}$	−0.003	0.084	0.956	−0.004	0.086	0.954	−0.002	0.063	0.988	−0.004	0.090	0.946
	$\theta_{5}$	−0.010	0.097	0.930	−0.005	0.098	0.934	−0.020	0.098	0.924	0.004	0.096	0.944
	$\theta_{6}$	−0.002	0.044	0.962	−0.001	0.045	0.956	−0.002	0.040	0.968	−0.001	0.046	0.948
	$\theta_{7}$	0.001	0.046	0.950	0.002	0.047	0.944	−0.001	0.045	0.948	0.004	0.047	0.946
	$\theta_{8}$	−0.001	0.043	0.960	−0.001	0.043	0.956	−0.001	0.039	0.970	−0.001	0.045	0.954
	$\theta_{9}$	−0.002	0.047	0.950	0.000	0.047	0.944	−0.005	0.048	0.942	0.002	0.047	0.952
	$\theta_{10}$	−0.004	0.044	0.958	−0.003	0.045	0.956	−0.003	0.039	0.962	−0.003	0.046	0.952
	$\beta_{1}$	−0.048	0.187	0.928	−0.025	0.184	0.936	−0.089	0.195	0.914	0.007	0.187	0.936
	$\beta_{2}$	−0.048	0.170	0.956	−0.028	0.166	0.958	−0.085	0.176	0.938	0.001	0.167	0.954
	$\beta_{3}$	−0.009	0.152	0.958	−0.007	0.159	0.950	−0.007	0.114	0.984	−0.007	0.172	0.936
	$\beta_{4}$	−0.003	0.143	0.960	−0.004	0.150	0.962	−0.002	0.104	0.988	−0.005	0.164	0.944
	$\beta_{5}$	−0.049	0.179	0.942	−0.027	0.174	0.958	−0.091	0.187	0.918	0.005	0.175	0.950
	$\beta_{6}$	−0.007	0.102	0.940	0.005	0.102	0.946	−0.031	0.104	0.928	0.020	0.107	0.944
	$\beta_{7}$	0.004	0.084	0.938	0.005	0.086	0.932	0.003	0.067	0.964	0.006	0.091	0.930
	$\beta_{8}$	−0.007	0.076	0.968	−0.007	0.078	0.962	−0.006	0.060	0.982	−0.008	0.082	0.952
	$\beta_{9}$	−0.011	0.099	0.958	0.001	0.098	0.958	−0.036	0.102	0.944	0.017	0.102	0.946
	$\beta_{10}$	0.006	0.082	0.950	0.006	0.084	0.944	0.005	0.065	0.984	0.006	0.088	0.934
	$\sigma_{u}^{2}$	0.002	0.088	0.964	0.006	0.091	0.956	−0.009	0.092	0.944	0.012	0.090	0.946
	$\sigma_{\xi }^{2}$	0.002	0.083	0.962	−0.001	0.083	0.954	0.009	0.085	0.944	−0.006	0.081	0.966
	$\sigma_{\epsilon }^{2}$	−0.001	0.036	0.952	−0.003	0.036	0.952	0.002	0.037	0.952	0.001	0.037	0.944
(0.5, 0.25)	$\theta_{1}$	−0.017	0.090	0.922	−0.011	0.088	0.926	−0.022	0.089	0.920	−0.001	0.088	0.926
	$\theta_{2}$	−0.005	0.076	0.960	−0.003	0.078	0.952	−0.004	0.046	0.994	0.000	0.083	0.942
	$\theta_{3}$	−0.016	0.084	0.940	−0.013	0.083	0.944	−0.017	0.083	0.938	−0.005	0.083	0.948
	$\theta_{4}$	−0.001	0.078	0.958	−0.001	0.079	0.956	−0.002	0.046	0.992	−0.002	0.085	0.942
	$\theta_{5}$	−0.019	0.087	0.934	−0.014	0.085	0.936	−0.024	0.087	0.920	−0.003	0.085	0.944
	$\theta_{6}$	−0.001	0.040	0.958	−0.001	0.040	0.966	−0.002	0.029	0.984	0.000	0.042	0.960
	$\theta_{7}$	−0.003	0.043	0.946	−0.002	0.043	0.944	−0.003	0.042	0.938	0.000	0.043	0.944
	$\theta_{8}$	−0.002	0.040	0.948	−0.002	0.040	0.950	−0.001	0.028	0.976	−0.002	0.042	0.946
	$\theta_{9}$	−0.008	0.043	0.948	−0.007	0.042	0.956	−0.009	0.043	0.950	−0.004	0.042	0.958
	$\theta_{10}$	0.003	0.039	0.952	0.003	0.039	0.952	0.003	0.028	0.980	0.004	0.041	0.944
	$\beta_{1}$	−0.040	0.178	0.944	−0.017	0.176	0.950	−0.074	0.182	0.926	0.017	0.178	0.946
	$\beta_{2}$	−0.032	0.177	0.940	−0.013	0.173	0.958	−0.064	0.175	0.922	0.017	0.179	0.946
	$\beta_{3}$	−0.011	0.154	0.956	−0.011	0.160	0.958	−0.009	0.117	0.982	−0.007	0.174	0.944
	$\beta_{4}$	−0.004	0.150	0.966	−0.005	0.156	0.958	−0.002	0.110	0.986	−0.006	0.171	0.948
	$\beta_{5}$	−0.051	0.186	0.932	−0.029	0.182	0.934	−0.086	0.192	0.916	0.005	0.181	0.948
	$\beta_{6}$	−0.008	0.104	0.936	0.003	0.104	0.938	−0.029	0.103	0.940	0.019	0.108	0.928
	$\beta_{7}$	−0.008	0.082	0.956	−0.007	0.084	0.958	−0.006	0.065	0.984	−0.006	0.088	0.954
	$\beta_{8}$	−0.002	0.081	0.956	−0.002	0.084	0.956	−0.001	0.064	0.970	−0.002	0.088	0.952
	$\beta_{9}$	−0.011	0.100	0.942	0.001	0.101	0.952	−0.032	0.103	0.938	0.018	0.103	0.944
	$\beta_{10}$	−0.001	0.076	0.964	−0.002	0.078	0.960	−0.002	0.060	0.978	−0.001	0.082	0.956
	$\sigma_{u}^{2}$	0.007	0.077	0.948	0.010	0.076	0.940	0.000	0.077	0.938	0.015	0.076	0.940
	$\sigma_{\xi }^{2}$	−0.003	0.075	0.936	−0.005	0.073	0.928	0.001	0.075	0.932	−0.009	0.073	0.932
	$\sigma_{\epsilon }^{2}$	0.002	0.009	0.952	0.001	0.009	0.954	0.002	0.010	0.944	0.002	0.009	0.948

**Table 3:** Simulation results in simulation Scenario I: 10 coeffients with 5 zeros. Summarized results include average size (aver.size): average number of coefficients labelled as “positive”, true negatives (TN): average number of coefficients labelled as “negative” correctly, and false negatives (FN): average number of coefficients labelled as “negative” incorrectly, for all parameter configurations for the longitudinal and survival submodels.

Longitudinal		Aver.size (%)				TN (%)				FN (%)			
$\sigma_{u}^{2}$	$\sigma_{\epsilon }^{2}$	BL	BAL	SS	GB	BL	BAL	SS	GB	BL	BAL	SS	GB
0.25	0.25	5.24	5.25	5.10	5.28	4.76	4.75	4.90	4.72	0.00	0.00	0.00	0.00
		(104.8)	(105.1)	(102.0)	(105.6)	(95.2)	(94.9)	(98.0)	(94.4)	(0.0)	(0.0)	(0.0)	(0.0)
	1	5.23	5.24	5.16	5.25	4.77	4.76	4.84	4.75	0.00	0.00	0.00	0.00
		(104.7)	(104.8)	(103.3)	(105.1)	(95.3)	(95.2)	(96.7)	(94.9)	(0.0)	(0.0)	(0.0)	(0.0)
0.5	0.25	5.22	5.22	5.07	5.27	4.78	4.78	4.93	4.73	0.00	0.00	0.00	0.00
		(104.5)	(104.5)	(101.5)	(105.3)	(95.5)	(95.5)	(98.5)	(94.7)	(0.0)	(0.0)	(0.0)	(0.0)
	1	5.19	5.21	5.13	5.24	4.81	4.79	4.87	4.76	0.00	0.00	0.00	0.00
		(103.8)	(104.3)	(102.5)	(104.9)	(96.2)	(95.7)	(97.5)	(95.1)	(0.0)	(0.0)	(0.0)	(0.0)
Survival		Aver.size (%)				TN (%)				FN (%)			
$\sigma_{u}^{2}$	$\sigma_{\epsilon }^{2}$	BL	BAL	SS	GB	BL	BAL	SS	GB	BL	BAL	SS	GB
0.25	0.25	5.20	5.22	5.10	5.27	4.80	4.78	4.90	4.73	0.00	0.00	0.00	0.00
		(104.0)	(104.4)	(102.0)	(105.4)	(96.0)	(95.6)	(98.0)	(94.6)	(0.0)	(0.0)	(0.0)	(0.0)
	1	5.24	5.25	5.11	5.31	4.76	4.75	4.89	4.69	0.00	0.00	0.00	0.00
		(104.7)	(105.0)	(102.2)	(106.2)	(95.3)	(95.0)	(97.8)	(93.8)	(0.0)	(0.0)	(0.0)	(0.0)
0.5	0.25	5.20	5.21	5.10	5.25	4.80	4.79	4.90	4.75	0.00	0.00	0.00	0.00
		(104.0)	(104.2)	(102.0)	(104.9)	(96.0)	(95.8)	(98.0)	(95.1)	(0.0)	(0.0)	(0.0)	(0.0)
	1	5.22	5.25	5.10	5.30	4.77	4.75	4.90	4.70	0.00	0.00	0.00	0.00
		(104.5)	(105.0)	(101.9)	(106.1)	(95.5)	(95.0)	(98.0)	(93.9)	(0.0)	(0.0)	(0.0)	(0.0)

**Table 4:** Simulation results for simulation Scenario II: 30 coefficients with 20 zeros. Summarized results include average size (aver.size), true negatives (TN), and false negatives (FN) for all parameter configurations for the longitudinal and survival submodels.

Longitudinal		Aver.size (%)				TN (%)				FN (%)			
$\sigma_{u}^{2}$	$\sigma_{\epsilon }^{2}$	BL	BAL	SS	GB	BL	BAL	SS	GB	BL	BAL	SS	GB
0.25	0.25	10.89	10.96	10.27	11.08	19.11	19.04	19.73	18.92	0.00	0.00	0.00	0.00
		(108.9)	(109.6)	(102.7)	(110.8)	(95.5)	(95.2)	(98.7)	(94.6)	(0.0)	(0.0)	(0.0)	(0.0)
	1	10.82	10.92	10.57	10.98	19.18	19.08	19.43	19.02	0.00	0.00	0.00	0.00
		(108.2)	(109.2)	(105.7)	(109.8)	(95.9)	(95.4)	(97.1)	(95.1)	(0.0)	(0.0)	(0.0)	(0.0)
0.5	0.25	10.91	10.96	10.23	11.14	19.09	19.04	19.77	18.86	0.00	0.00	0.00	0.00
		(109.1)	(109.6)	(102.3)	(111.4)	(95.5)	(95.2)	(98.9)	(94.3)	(0.0)	(0.0)	(0.0)	(0.0)
	1	10.84	10.90	10.50	10.97	19.16	19.10	19.50	19.03	0.00	0.00	0.00	0.00
		(108.4)	(109.0)	(105.0)	(109.7)	(95.8)	(95.5)	(97.5)	(95.1)	(0.0)	(0.0)	(0.0)	(0.0)
Survival		Aver.size (%)				TN (%)				FN (%)			
$\sigma_{u}^{2}$	$\sigma_{\epsilon }^{2}$	BL	BAL	SS	GB	BL	BAL	SS	GB	BL	BAL	SS	GB
0.25	0.25	10.08	10.64	8.66	11.12	19.26	18.90	19.81	18.53	0.66	0.46	1.53	0.35
		(100.8)	(106.4)	(86.6)	(111.2)	(96.3)	(94.5)	(99.0)	(92.6)	(3.3)	(2.3)	(7.7)	(1.8)
	1	10.03	10.61	8.50	11.22	19.29	18.90	19.78	18.38	0.68	0.49	1.72	0.40
		(100.3)	(106.1)	(85.0)	(112.2)	(96.5)	(94.5)	(98.9)	(91.9)	(3.4)	(2.5)	(8.6)	(2.0)
0.5	0.25	9.89	10.47	8.25	11.04	19.31	18.98	19.84	18.56	0.80	0.55	1.91	0.40
		(98.9)	(104.7)	(82.5)	(110.4)	(96.6)	(94.9)	(99.2)	(92.8)	(4.0)	(2.8)	(9.6)	(2.0)
	1	9.81	10.45	8.14	10.95	19.32	18.93	19.82	18.56	0.87	0.61	2.04	0.49
		(98.1)	(104.5)	(81.4)	(109.5)	(96.6)	(94.7)	(99.1)	(92.8)	(4.3)	(3.1)	(10.2)	(2.4)

**Table 5:** Posterior means and corresponding 95% credible intervals for the covariate effects on the longitudinal response in the real data analysis. The covariates are Female $\left(X_{1}\right)$, Initial BMI $\left(X_{2}\right)$, Parental history of high blood pressure $\left(X_{3}\right)$, Depression $\left(X_{4}\right)$, Anxiety $\left(X_{5}\right)$, Smoking $\left(X_{6}\right)$, Heavy drinking $\left(X_{7}\right)$, Married $\left(X_{8}\right)$, Inactive $\left(X_{9}\right)$, Initial age $\left(X_{10}\right)$, Parent history of diabetes $\left(X_{11}\right)$, Glucose $\left(X_{12}\right)$, Triglyceride $\left(X_{13}\right)$, Fatigue $\left(X_{14}\right)$, Night sweats $\left(X_{15}\right)$, Any type of cancer $\left(X_{16}\right)$, Chest pain or pressure $\left(X_{17}\right)$, Heart attack $\left(X_{18}\right)$, Rapid or irregular heartbeats $\left(X_{19}\right)$, Fainting or lightheadedness $\left(X_{20}\right)$, Rheumatic fever $\left(X_{21}\right)$, Calf pain with exercise $\left(X_{22}\right)$, Varicose veins $\left(X_{23}\right)$, Thyroid disease $\left(X_{24}\right)$, Parent history of premature coronary heart disease or stroke $\left(X_{25}\right)</Table\_Caption>$

	BL	BAL	SS	GB
$X_{1}$	**0.12(0.08,0.16)**	**0.12(0.08,0.16)**	**0.12(0.08,0.15)**	**0.14(0.10,0.18)**
$X_{2}$	0.01(−0.00,0.03)	0.01(−0.00,0.03)	0.01(−0.003,0.03)	0.01(−0.002,0.03)
$X_{3}$	**-0.13(−0.17,−0.09)**	**-0.13(−0.17,−0.09)**	**-0.12(−0.16,−0.09)**	**-0.13(−0.17,−0.09)**
$X_{4}$	−0.04(−0.12,0.04)	−0.04(−0.13,0.04)	−0.03(−0.11,0.04)	−0.04(−0.12,0.04)
$X_{5}$	0.00(−0.07,0.07)	0.00(−0.07,0.07)	−0.01(−0.07,0.06)	0.00(−0.08,0.07)
$X_{6}$	0.03(−0.01,0.07)	0.03(−0.01,0.07)	0.03(−0.01,0.06)	**0.05(0.01,0.08)**
$X_{7}$	**0.13(0.08,0.18)**	**0.13(0.08,0.18)**	**0.12(0.07,0.16)**	**0.14(0.09,0.19)**
$X_{8}$	**-0.31(−0.35,−0.28)**	**-0.31(−0.35,−0.28)**	**-0.31(−0.34,−0.28)**	**-0.29(−0.32,−0.26)**
$X_{9}$	**0.06(0.03,0.10)**	**0.06(0.03,0.09)**	**0.06(0.03,0.09)**	**0.08(0.05,0.12)**
$X_{10}$	**0.24(0.22,0.26)**	**0.24(0.22,0.26)**	**0.24(0.22,0.25)**	**0.24(0.22,0.25)**
$X_{11}$	**-0.06(−0.11,−0.00)**	−0.06(−0.11,0.00)	**-0.05(−0.11,−0.00)**	−0.06(−0.11,0.00)
$X_{12}$	−0.00(−0.02,0.01)	−0.001(−0.01,0.01)	0.000(−0.01,0.01)	−0.00(−0.01,0.01)
$X_{13}$	**0.20(0.19,0.22)**	**0.20(0.19,0.22)**	**0.20(0.19,0.22)**	**0.20(0.19,0.21)**
$X_{14}$	−0.01(−0.08,0.06)	−0.02(−0.09,0.06)	−0.01(−0.08,0.05)	−0.01(−0.09,0.06)
$X_{15}$	−0.05(−0.14,0.04)	−0.05(−0.14,0.04)	−0.04(−0.12,0.04)	−0.05(−0.14,0.04)
$X_{16}$	−0.08(−0.16,−0.00)	**-0.08(−0.16,−0.00)**	−0.07(−0.14,0.00)	−0.08(−0.16,0.00)
$X_{17}$	**0.04(0.00,0.08)**	**0.04(0.00,0.08)**	**0.04(0.00,0.07)**	**0.05(0.02,0.09)**
$X_{18}$	−0.10(−0.21,0.02)	−0.10(−0.21,0.02)	−0.07(−0.17,0.03)	−0.09(−0.20,0.03)
$X_{19}$	0.00(−0.04,0.04)	0.00(−0.04,0.04)	0.00(−0.03,0.04)	0.01(−0.02,0.05)
$X_{20}$	−0.04(−0.11,0.03)	−0.03(−0.11,0.04)	−0.03(−0.10,0.04)	−0.04(−0.11,0.03)
$X_{21}$	−0.00(−0.09,0.09)	0.00(−0.10,0.10)	−0.00(−0.09,0.08)	0.01(−0.08,0.11)
$X_{22}$	−0.00(−0.08,0.08)	−0.00(−0.08,0.08)	−0.00(−0.08,0.07)	0.00(−0.08,0.08)
$X_{23}$	−0.07(−0.18,0.03)	−0.08(−0.19,0.03)	−0.06(−0.16,0.03)	−0.08(−0.19,0.03)
$X_{24}$	0.00(−0.06,0.07)	0.00(−0.06,0.06)	0.01(−0.05,0.07)	0.01(−0.05,0.08)
$X_{25}$	**0.07(0.04,0.10)**	**0.07(0.04,0.10)**	**0.07(0.04,0.10)**	**0.09(0.07,0.12)**

**Table 6:** Posterior means and corresponding 95% credible intervals for the covariate effects on the survival response in the real data analysis.

	BL	BAL	SS	GB
$X_{1}$	**-0.30(−0.36,−0.23)**	**-0.30(−0.36,−0.24)**	**-0.29(−0.35,−0.22)**	**-0.30(−0.36,−0.23)**
$X_{2}$	**0.25(0.22,0.27)**	**0.25(0.22,0.27)**	**0.25(0.22,0.27)**	**0.25(0.22,0.27)**
$X_{3}$	**0.30(0.23,0.36)**	**0.30(0.24,0.37)**	**0.29(0.22,0.35)**	**0.30(0.24,0.37)**
$X_{4}$	−0.04(−0.17,0.08)	−0.05(−0.18,0.08)	−0.04(−0.15,0.08)	−0.05(−0.18,0.08)
$X_{5}$	0.06(−0.06,0.17)	0.06(−0.05,0.18)	0.05(−0.05,0.15)	0.06(−0.06,0.18)
$X_{6}$	−0.05(−0.10,0.01)	−0.05(−0.11,0.01)	−0.04(−0.10,0.01)	−0.05(−0.11,0.01)
$X_{7}$	**0.19(0.12,0.27)**	**0.19(0.12,0.27)**	**0.18(0.10,0.26)**	**0.20(0.12,0.27)**
$X_{8}$	**-0.07(−0.12,−0.02)**	**-0.07(−0.12,−0.02)**	**-0.06(−0.11,−0.01)**	**-0.07(−0.12,−0.02)**
$X_{9}$	0.05(−0.00,0.09)	**0.05(0.00,0.09)**	0.05(−0.00,0.09)	**0.05(0.00,0.10)**
$X_{10}$	**-0.41(−0.44,−0.39)**	**-0.42(−0.44,−0.39)**	**-0.41(−0.44,−0.39)**	**-0.41(−0.44,−0.38)**
$X_{11}$	−0.05(−0.13,0.04)	−0.05(−0.13,0.04)	−0.04(−0.12,0.04)	−0.05(−0.13,0.04)
$X_{12}$	**-0.03(−0.06,−0.01)**	**-0.03(−0.06,−0.01)**	**-0.03(−0.06,−0.01)**	**-0.03(−0.06,−0.01)**
$X_{13}$	**0.11(0.09,0.14)**	**0.12(0.09,0.14)**	**0.11(0.09,0.14)**	**0.11(0.09,0.14)**
$X_{14}$	−0.01(−0.13,0.10)	−0.01(−0.13,0.10)	−0.01(−0.12,0.09)	−0.01(−0.13,0.11)
$X_{15}$	0.01(−0.12,0.15)	0.02(−0.13,0.16)	0.01(−0.12,0.13)	0.02(−0.13,0.16)
$X_{16}$	0.09(−0.03,0.21)	0.09(−0.03,0.21)	0.07(−0.04,0.19)	0.09(−0.03,0.22)
$X_{17}$	**0.07(0.01,0.12)**	**0.07(0.01,0.12)**	**0.07(0.01,0.12)**	**0.07(0.01,0.13)**
$X_{18}$	−0.01(−0.18,0.16)	−0.01(−0.19,0.16)	−0.01(−0.15,0.14)	−0.01(−0.20,0.17)
$X_{19}$	**0.10(0.04,0.16)**	**0.10(0.05,0.16)**	**0.10(0.04,0.16)**	**0.11(0.05,0.17)**
$X_{20}$	−0.08(−0.19,0.03)	−0.08(−0.20,0.03)	−0.07(−0.18,0.03)	−0.09(−0.21,0.03)
$X_{21}$	0.00(−0.14,0.14)	0.00(−0.15,0.14)	0.00(−0.12,0.13)	0.00(−0.15,0.15)
$X_{22}$	0.03(−0.09,0.15)	0.03(−0.10,0.16)	0.02(−0.09,0.13)	0.03(−0.10,0.16)
$X_{23}$	−0.05(−0.22,0.12)	−0.05(−0.23,0.12)	−0.04(−0.18,0.11)	−0.06(−0.23,0.12)
$X_{24}$	0.01(−0.09,0.10)	0.007(−0.09,0.11)	0.004(−0.09,0.10)	0.009(−0.09,0.11)
$X_{25}$	**0.08(0.04,0.13)**	**0.09(0.04,0.13)**	**0.08(0.04,0.13)**	**0.09(0.04,0.13)**

**Table 7:** Posterior means and corresponding 95% credible intervals for two frailty variances $\sigma_{u}^{2}$ and $\sigma_{\xi }^{2}$, error variance $\sigma_{\epsilon }^{2}$, and association parameter τ from the proposed methods when using different number of interior knots $\left(\tilde{J},J^{*}\right)$ in the monotone spline specifications.

$\left(\tilde{J},J^{*}\right)$		BL	BAL	SS	GB
(3, 3)	$\sigma_{u}^{2}$	0.043(0.030, 0.057)	0.045(0.034, 0.057)	0.041(0.028, 0.060)	0.040(0.025, 0.056)
	$\sigma_{\xi }^{2}$	0.437(0.418, 0.456)	0.435(0.418, 0.452)	0.439(0.417, 0.459)	0.441(0.421, 0.461)
	$\sigma_{\epsilon }^{2}$	0.498(0.493, 0.504)	0.498(0.493, 0.504)	0.498(0.493, 0.504)	0.498(0.493, 0.504)
	τ	−0.027(−0.036, −0.019)	−0.028(−0.036, −0.022)	−0.025(−0.034, −0.016)	−0.025(−0.035, −0.016)
(6, 6)	$\sigma_{u}^{2}$	0.043(0.032, 0.057)	0.042(0.029, 0.060)	0.046(0.032, 0.060)	0.041(0.030, 0.054)
	$\sigma_{\xi }^{2}$	0.438(0.419, 0.455)	0.438(0.417, 0.456)	0.434(0.416, 0.453)	0.440(0.421, 0.458)
	$\sigma_{\epsilon }^{2}$	0.498(0.493, 0.504)	0.498(0.493, 0.504)	0.498(0.493, 0.504)	0.498(0.493, 0.504)
	τ	−0.027(−0.036, −0.020)	−0.027(−0.037, −0.019)	−0.028(−0.037, −0.019)	−0.026(−0.034, −0.019)
(8, 8)	$\sigma_{u}^{2}$	0.042(0.029, 0.058)	0.042(0.030, 0.058)	0.043(0.029, 0.061)	0.045(0.034, 0.058)
	$\sigma_{\xi }^{2}$	0.438(0.418, 0.457)	0.438(0.418, 0.457)	0.437(0.417, 0.457)	0.436(0.418, 0.454)
	$\sigma_{\epsilon }^{2}$	0.498(0.493, 0.504)	0.498(0.493, 0.504)	0.498(0.493, 0.504)	0.498(0.493, 0.504)
	τ	−0.027(−0.036, −0.018)	−0.027(−0.036, −0.019)	−0.026(−0.035, −0.019)	−0.028(−0.036, −0.021)
